# Screening and characterization of *Bacillus velezensis* LB-Y-1 toward selection as a potential probiotic for poultry with multi-enzyme production property

**DOI:** 10.3389/fmicb.2023.1143265

**Published:** 2023-04-17

**Authors:** Chong Li, Shuzhen Li, Guoqi Dang, Rui Jia, Si Chen, Xuejuan Deng, Guohua Liu, Yves Beckers, Huiyi Cai

**Affiliations:** ^1^Key Laboratory for Feed Biotechnology of the Ministry of Agriculture and Rural Affairs, Institute of Feed Research, Chinese Academy of Agriculture Sciences, Beijing, China; ^2^Precision Livestock and Nutrition Laboratory, Teaching and Research Centre (TERRA), Gembloux Agro-Bio Tech, University of Liège, Gembloux, Belgium; ^3^Department of Molecular Cell Biology, Samsung Medical Center, Sungkyunkwan University School of Medicine, Suwon, Republic of Korea; ^4^National Engineering Research Center of Biological Feed, Beijing, China

**Keywords:** *Bacillus velezensis*, multi-enzyme, broiler chickens, tibia mineralization, digestive enzymes, intestinal microbiota

## Abstract

*Bacillus* spp. have gained increasing recognition as an option to use as antimicrobial growth promoters, which are characterized by producing various enzymes and antimicrobial compounds. The present study was undertaken to screen and evaluate a *Bacillus* strain with the multi-enzyme production property for poultry production. LB-Y-1, screened from the intestines of healthy animals, was revealed to be a *Bacillus velezensis* by the morphological, biochemical, and molecular characterization. The strain was screened out by a specific screening program, possessed excellent multi-enzyme production potential, including protease, cellulase, and phytase. Moreover, the strain also exhibited amylolytic and lipolytic activity *in vitro*. The dietary LB-Y-1 supplementation improved growth performance and tibia mineralization in chicken broilers, and increased serum albumin and serum total protein at 21 days of age (*p* < 0.05). Besides, LB-Y-1 enhanced the activity of serum alkaline phosphatase and digestive enzyme in broilers at 21 and 42 days of age (*p* < 0.05). Analysis of intestinal microbiota showed that a higher community richness (Chao1 index) and diversity (Shannon index) in the LB-Y-1 supplemented compared with the CON group. PCoA analysis showed that the community composition and structure were distinctly different between the CON and LB-Y-1 group. The beneficial genera such as *Parasutterella* and *Rikenellaceae* were abundant, while the opportunistic pathogen such as *Escherichia-Shigella* were reduced in the LB-Y-1 supplemented group (*p* < 0.05). Collectively, LB-Y-1 can be considered as a potential strain for further utilization in direct-fed microbial or starter culture for fermentation.

## 1. Introduction

“Beneficial,” “friendly,” and “healthy” are commonly used to describe probiotics. Previous studies have proved that probiotics may have a fundamental impact on the maturation of animal immune phenotype, and the metabolisms of different functional microorganisms would play a crucial role in the maintenance of health (Gao et al., 2017; Ma et al., 2018). Therefore, it is imperative to screen functional microorganisms with excellent characteristics and explore their roles in different periods of life (Kundu et al., 2017; Francino, 2018; Tun et al., 2018). One of the functional properties of microorganism is the ability to secrete multi-enzyme by exocytosis, and these enzymes are able to further degrade certain nutrient substances that are not well digested by endogenous enzymes produced in the animal’s digestive system (Lee et al., 2020). For example, the proteases can convert insoluble storage protein into soluble peptides and amino acids (Kesari and Rangan, 2011). The cellulases such as cellobiohydrolases, β-glucosidases, and endoglucanases can degrade the fiber components in food (Stålbrand et al., 1998; Ye et al., 2017). Microbial phytases can hydrolyze the phytate complex and release the nutrients for use by the broilers (Babatunde et al., 2021; Li et al., 2022).

One of the main objectives in the poultry industry is to maximize the productivity. However, several major issues confront the poultry meat production today, such as the imbalance in protein resources, and viral or bacterial infectious diseases. In parallel, the global trend for reducing antibiotic growth promoters in the animal production has gathered momentum (Pourabedin et al., 2015). It is particularly important to explore the nutritional interventions for the prevention of these pathological processes, and the supplementation of exogenous amylase, protease, or other enzymes for broiler diets could be a strategic approach (Chang’a et al., 2019; Córdova-Noboa et al., 2021). Studies have shown that a number of *Bacillus* spp. can be added to feed, which has the effect of improving animal growth performance, regulating the intestinal micro-ecology, and promoting the utilization of nutrients (Lee et al., 2008; Li et al., 2022).

Researchers have confirmed that the *Bacillus velezensis* (*B. velezensis*) has the characteristics of producing a variety of enzymes like protease, cellulase, amylase, and glucanase, as well as secreting antibacterial substances to inhibit the growth of pathogenic microorganisms (Meng and Hao, 2017; Li et al., 2018). However, a comprehensive evaluation of the effect of *B. velezensis* on poultry is lacking. In the current study, *B. velezensis* LB-Y-1 with the ability to produce multi-enzyme including protease, cellulase, and phytase was screened and characterized from the intestinal tract of different healthy animals. Ultimately, the strain was comprehensively evaluated for its safety and efficacy in growing broilers.

## 2. Materials and methods

### 2.1. Sample collection and isolation of *Bacillus* spp.

The strains were isolated from the digestive tracts of healthy and free-ranging animals (the rumen of cattles, and the cecum of chickens, pigs, and rabbits) without any additives during the rearing period (such as antibiotics or probiotics). All strains were collected in accordance with the Bioconvention and the Nagoya protocol (Diversity, 2022). *Bacillus* spp. was isolated according to the method described as previously, with minor modification (Susanti et al., 2021; Liu et al., 2022). Heat the intestinal contents at 95°C for 5 min in order to separate out the *Bacillus* spores, followed by a 10-fold series dilution with sterile normal saline to ensure recovery of individual colonies on agar plates. Isolation was performed using agar spot test on Luria-Bertani (LB)—casein medium (Hope Bio-Technology Co., Ltd., Qingdao, China; casein, 0.4%). Based on the colony morphology and casein-soluble region of *Bacillus* spp., 191 colonies were purified and selected. The strains were grown in LB broth (Hope Bio-Technology Co., Ltd., Qingdao, China) and stored in LB broth with 20% glycerol at −80°C.

### 2.2. Protease production capacity (primary screening)

The protocol was based on the previous method with minor modification (Liu et al., 2022). Briefly, the selected strains were cultured at 37°C for 24 h and the inoculum was added at 10% (v/v) rate to the fermentation broth containing 20 g peanut meal and 20 ml distilled water in a flask. The cultures were incubated anaerobically at 37°C for 72 h. The fermentation product (10 g) was added to a 250 mL Erlenmeyer flask containing 90 ml of distilled water and incubated in 40°C water bath for 1 h (stirred every 15 min). The crude enzyme solution was obtained after filtration through filter paper Whatman No.1 (Whatman International Ltd., Maidstone, United Kingdom). Folin–Ciocalteu method was used to determine the protease activity (Ramkumar et al., 2018). All tests were repeated four times.

### 2.3. Cellulase production capacity (secondary screening)

The cellulase-production strains were screened by the carboxymethylcellulose (CMC) plate assay method (Shaikh et al., 2013; Mohammad et al., 2017). In brief, the plates were incubated at 37°C for 5 days to produce enough cellulase, then the plates were incubated in Congo red solution (1% w/v) for 15 min at 37°C, followed by flooding with 1 M NaCl for 15 min. The strains with cellulose degrading ability showed a clear zone. The ratio of clear zone diameter to colony diameter (mm/mm) was considered as degradation efficiency. All tests were repeated four times.

### 2.4. Phytase production capacity (tertiary screening)

The phytase-production strains were screened following the method explained by Demirkan et al. (2014). Firstly, the freshly cultured strains in LB broth (OD_600_ = 0.3) were inoculated into enzyme production broth [Dextrose 0.5%, yeast extract 0.5%, peptone 1.0%, sodium phytate 0.1%, CaCl_2_ 0.1%, MgSO_4_ 0.1% (w/v, pH 7.0)] at 1% rate, and then incubated at 37°C, 150 rpm for 24, 32, 48, 56, 72, and 80 h in a shaking incubator. At the end of each period, the culture supernatant was collected for determination of phytase activity using the method explained by Choi et al. (2001). All tests were repeated four times, and selected the strains with high phytase activity for further analysis.

### 2.5. Morphological, biochemical, and molecular characterization of LB-Y-1

The characterizations of LB-Y-1 were done using the method explained by Zhang D. F. et al. (2021) with minor modifications. The characteristic colonies were gram stained, and bacteria were examined for morphology under a light microscope (Olympus CX40, Olympus Optical Co. Ltd., Hamburg, Germany) and a scanning electron microscopic (SEM, Inspect F50, FEI Company, Hillsboro, OR, United States; Bajpai et al., 2016). The growth curve assay of LB-Y-1 was performed according to the method described previously (Fitzgerald et al., 2020). Supplementary File 1 showed the molecular biology identification procedure for LB-Y-1. A bootstrap phylogenetic tree was constructed by the neighbor-joining method, using MEGA 7 software.^1^ Furthermore, biochemical characteristics of LB-Y-1 were performed using the API 50 CHB system (BioMerieux Vitek, France) according to the instructions from the manufacturer.

In order to further examine the potential of LB-Y-1 to produce multi-enzymes, the activity of amylase and lipase in the LB-Y-1 were determined using the solid agar plate method (Zhang D. F. et al., 2021). Briefly, after cultured in LB broth at 37°C overnight, the strain was stab-inoculated on the surface of LB agar plate containing 1.5% soluble starch or 1% triglyceride tributyrate (four replicates were made of each assay). The plates were subsequently incubated at 37°C for 24 h. The amylase and lipase activities were determined based on the diameter of the transparent halos.

### 2.6. Safety evaluation of LB-Y-1 (*in vitro*)

#### 2.6.1. Hemolytic activity assay

The hemolytic activity of LB-Y-1 was determined using the method explained by Maragkoudakis et al. (2006), with minor modifications. Briefly, LB-Y-1 was cultured in LB broth overnight, and then inoculated in pre-made blood agar (Beijing Land Bridge Technology Co., Ltd., Beijing, China) containing 5% (v/v) sheep blood. The presence of a hemolysis zone was observed following incubation at 37°C for 24 h.

#### 2.6.2. Antibiotic susceptibility

Antibiotic susceptibility of LB-Y-1 was determined by the agar diffusion method of CLSI (2015), with minor modifications. Briefly, LB-Y-1 was cultured in LB broth at 37°C for 12 h, and then preparing a suspension in accordance with two McFarland’s scales (10^8^ CFU/mL; Reuben et al., 2019). A total of 100 μL suspension were spread onto MRS agar plates, in which the antibiotic discs were placed. The commercial antibiotic discs (HANGWEI, Hangzhou, China) contains Cefoperazone (75 μg), Ceftriaxone (30 μg), Ceftazidime (30 μg), Cefuroxime (30 μg), Cefradine (30 μg), Cefazolin (30 μg), Cefalexin (30 μg), Minocycline (30 μg), Doxycycline (30 μg), Tetracycline (30 μg), Ciprofloxacin (5 μg), Clindamycin (2 μg), Erythromycin (15 μg), Neomycin (30 μg), Kanamycin (30 μg), Gentamicin (10 μg), Amikacin (30 μg), Vancomycin (30 μg), Piperacillin (100 μg), Ampicillin (100 μg), Oxacillin (1 μg), Penicillin (10 μg), Chloramphenicol (30 μg), and Furazolidone (300 μg). Plates were incubated for 24 h at 37°C and the diameters of the clear zones were measured and classified as sensitive (S), intermediate (I), and resistance (R) according to the guidelines for CLSI.

### 2.7. *In vivo* testing

#### 2.7.1. Strain preparation

The strain *B. velezensis* LB-Y-1 was emulsified into microcapsules (prepared by Challenge Biotechnology Co., LTD, Beijing, China, viable count ≥ 5.0 × 10^10^ CFU/g). Following a conservative strategy, the amount of LB-Y-1 in feed was examined daily throughout the experiment to ensure cell viability (Nikoskelainen et al., 2003).

#### 2.7.2. Experimental design and bird management

The research was conducted at Nankou pilot base of the Chinese Academy of Agricultural Sciences (CAAS). The animal experiments were performed under the National Institute of Animal Health approved protocol and the ARRIVE guidelines were followed for reporting results (Kilkenny et al., 2012).

A total of 120 one-day-old male Arbor Acres (AA) broiler chickens (body weight, 40.2 ± 0.4 g) were randomly allocated into 2 treatment groups with 6 replicates of 10 birds each replicate. The control (CON) group were fed a corn-soybean-based diet, which met the nutritional requirements of broilers (Supplementary Table S1) in pellet form. LB-Y-1 homogenate was added at 100 mg/kg to the basal diets (BV group), and the final concentration was 3.5 × 10^9^ CFU/kg. The mixing and pelleting were operated by a single trained person and fresh diets were produced every 3 days to ensure uniformity of additives. The experiment lasted for 42 days in two feeding phases, starter (1–21 days) and grower (22–42 days), and all the broilers were housed in the same environmentally controlled facility (cleaning cage equipped with the fiberglass feeders and plastic net floor). All broilers were allowed *ad libitum* feed and water, and given the same photoperiod (16 h light: 8 h dark), relative humidity (1–7 days, 60%–70%; 8–42 days, 50%–60%) and room temperature (1–7 days, 33°C ± 2°C; 8–16 days decreased stepwise to 24°C; 17–42 days 24°C). The excreta was cleared daily. Broilers were subjected a routine vaccination program, and their health was monitored daily.

#### 2.7.3. Sampling

Body weight (BW) were measured at 21 and 42 days of age and record the feed intake, average daily feed intake (ADFI), average daily gain (ADG), and the feed intake/weight gain (F/G) ratio were calculated for the different phases. At 21 and 42 days of age, one bird close to the average body weight from each replicate was selected after 12 h fasting. Blood samples (2.5 mL) were collected from the wing vein in the EDTA-containing (5 mL). Serum was harvested from non-anticoagulated whole blood by centrifuging at 3,000 × *g* for 10 min, and stored at −20°C until analyzed. The slaughter performance and immune organ indexes were measured according to the production performance noun terms and metric statistics method of poultry (NY/T823-2004; Liu et al., 2021; Gao et al., 2022). One side of the tibia bone were removed for mineralization analysis. The jejunum digesta samples were collected from the middle of the jejunum and stored at −20°C until further analysis. Liver, spleen, and intestinal tissues (jejunum and ileum) were collected and fixed in 10% buffered formaldehyde (pH 7.4) for histological analysis. At 42 days of age, the ileal contents of 6 broilers were collected and snap-frozen in liquid nitrogen, followed by storage at −80°C for DNA extraction.

#### 2.7.4. Hematological and serum biochemical indexes analysis

Using an auto hematology analyzer (Sysme XT-1800i, China) to detect red blood cell count (RBC), white blood cell count (WBC), lymphocytes (LYM), and hemoglobin concentration (HGB). The alkaline phosphatase (ALP), aspartate aminotransferase (AST), alanine aminotransferase (ALT), and P level were measured using an automatic blood biochemical analyzer (Olympus AU640, Japan). The total protein (TP) and albumin levels of serum were measured using commercial assay kits (Nanjing Jiancheng Bioengineering Institute, Nanjing, China) by colorimetric method. Because the TP in serum mainly consists of albumin and globulin, the globulin content was obtained by subtracting the albumin value from that of the TP (Salem et al., 2018).

#### 2.7.5. Intestinal digestive enzyme and tibia bone mineralization analysis

Amylase, trypsin, and lipase activities of the digesta samples were measured by using the commercial assay kits (C016, A080 and A054, Nanjing Jiancheng Bioengineering Institute, Nanjing, China). Tibia bone mineralization analysis involved measuring ash of tibia bone and P and Ca concentration in the bone ash, according to the method reported by Li et al. (2022).

#### 2.7.6. Histological analysis

Fixed intestine samples were dehydrated in various concentrations of ethyl alcohol (75%, 85%, 95%, and 100%), cleared in xylene, and embedded in paraffin. Five micrometer-thick sections were prepared using a microtome, then the paraffin-embedded sections were deparaffinized, dehydrated, and stained by hematoxylin–eosin (H&E). Visualization was performed under a light microscope (Olympus CX40, Olympus Optical Co. Ltd., Hamburg, Germany). For the jejunum and ileum sections, epithelial thickness, villus length, and crypt depth were measured at least 10 well-oriented villi, and the villus length/crypt depth ratio (V/C) ratio was calculated.

#### 2.7.7. Microbial analysis

Total genomic DNA was extracted from the intestinal contents using commercially kit (Qiagen, Hilden, Germany), and the quality of DNA was checked by agarose gel electrophoresis. DNA concentration and purity were investigated using a NanoDrop2000 spectrophotometer (Thermo Fisher Scientific, Waltham, United States). The V3–V4 hypervariable regions from 16S rRNA gene was amplified with universal primers 338F (ACTCCTACGGGAGGCAGCAG) and 806R (GGACTACHVGGGTWTCTAAT). The amplicon was PCR purified and quantified as previously described (Takeshita et al., 2016). The library preparation and sequencing were carried out by Majorbio Biotech Co., Ltd. (Shanghai, China) using the Illumina MiSeq platform (Illumina, Madison, United States). The original data were subjected to quality control and species annotation processes to obtain effective Tags. Sequences were clustered into operational taxonomic units (OTUs) with 97% consistency, diversity and taxonomy was obtained by using QIIME software (version 1.9.1). Data were analyzed using the online platform of Majorbio Technology Co., Ltd.^2^ The alpha-diversity analysis was based on the normalized data including Chao1 index, Shannon index, Coverage index and numbers of OUTs. The beta-diversity was presented using principal coordinate analysis (PCoA; Lozupone and Knight, 2005). The LefSe (linear discriminant analysis effect size) analysis was performed using LefSe software (Segata et al., 2011). The relative abundance of the relative abundance at the phylum and genus levels were analyzed by the Student’s *t*-test.

### 2.8. Statistical analysis

All experimental data were tested for normality by using the Shapiro–Wilk normality test and for homogeneity of variances by using Levene’s test of SPSS19.0 software package for Windows (SPSS Inc., Chicago, IL, United States). Afterward, the data were analyzed by student’s *t*-test or one-factor analysis of variance (ANOVA) where appropriate. *p* < 0.05 and *p* < 0.01 showed a statistically significant and highly significant. For the indexes expressed as means with standard error of mean (SEM).

## 3. Results

**Figure 1:**
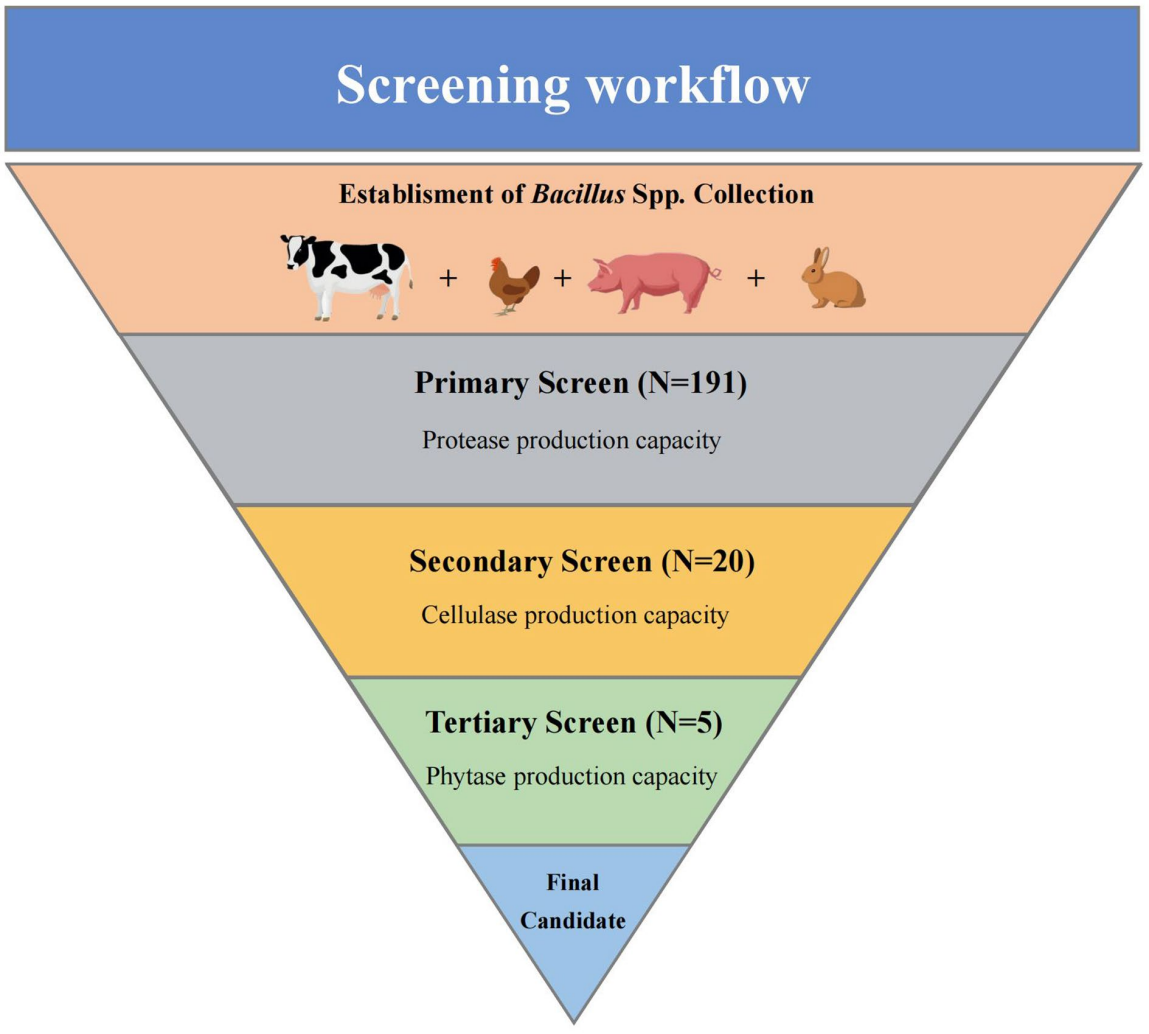
Workflow of the screening. The process was divided into three parts and performed to narrow down a total of 191 *Bacillus* spp. isolates to one strain with the ability to produce multi-enzyme.

A total of 191 potential *Bacillus* spp. were isolated. The screening process was composed of three steps, which was enabled to detect *Bacillus* spp. with the best multi-enzyme production properties from all candidates, the workflow is given in Figure 1.

### 3.1. Protease production capacity (primary screening)

**Figure 2:**
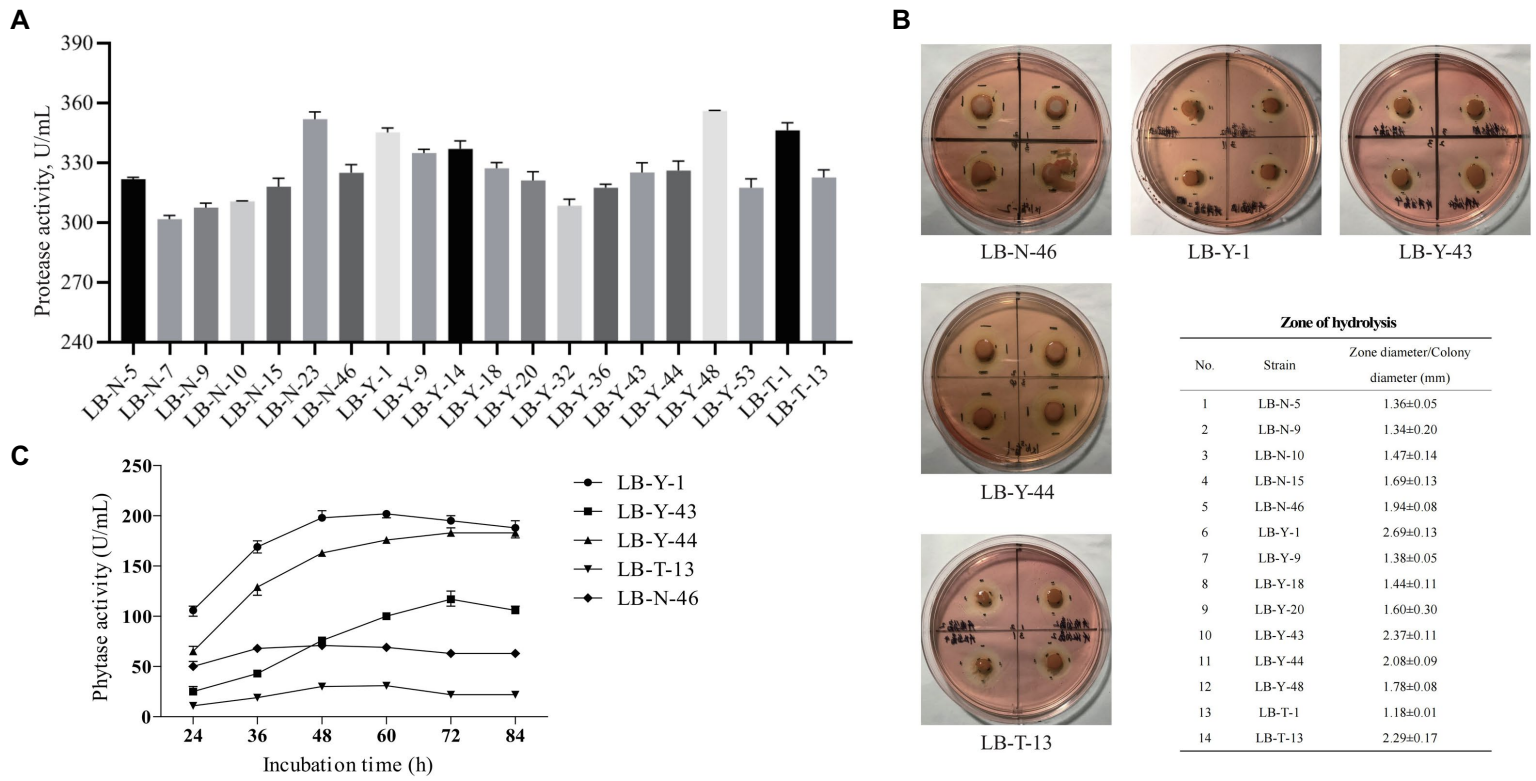
The screening process of *Bacillus* spp. **(A)** Protease production capacity (Primary Screen). **(B)** Cellulase production capacity (Secondary Screen). **(C)** Phytase production capacity (Tertiary Screen). Values are presented as Mean ± SEM.

In the primary screening, potential strains with strong protease production capacity were selected, and the protease activity depended on source of origin and the specific strain. The best 20 isolates with superior protease production potential that are depicted in Figure 2A were further screened.

### 3.2. Cellulase production capacity (secondary screening)

After the primary screening, the selected *Bacillus* spp. were tested for cellulase production capacity. A total of 14 strains were screened through the CMC plate assay method (Figure 2B), among which, 5 strains were found to have the largest degradation zone. Therefore, these strains (LB-N-46, LB-Y-1, LB-Y-43, LB-Y-44, and LB-T-13) were the best candidates for cellulase production capacity, and were further analyzed.

### 3.3. Phytase production capacity (tertiary screening)

The capacity of phytase production of the candidates is depicted in Figure 2C. Strains LB-Y-43, LB-T-13, and LB-N-46 exhibited low phytase activity during the culture, while LB-Y-1 and LB-Y-44 showed a higher activity, and LB-Y-1 reached the highest enzyme activity at earlier time point (48 h). Through the screening and assessment process, LB-Y-1 was selected as a potential strain for subsequent experiments.

### 3.4. Morphological, biochemical, and molecular characterization of LB-Y-1

**Figure 3:**
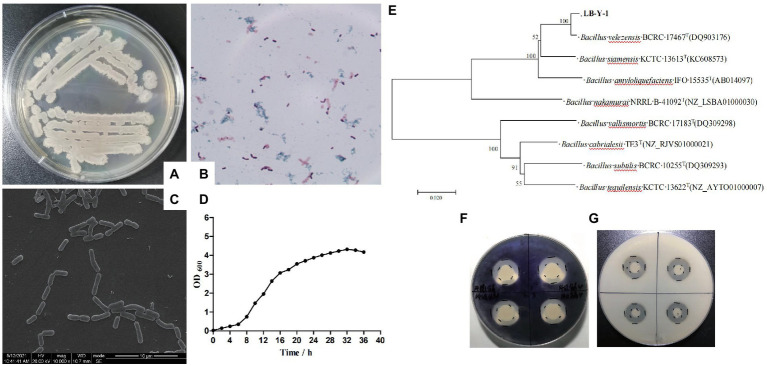
Morphological, biochemical, and molecular characteristics of LB-Y-1. **(A)** Colony morphology. **(B)** Gram stain showing Gram-positive rod. **(C)** SEM image of LB-Y-1 showing a small rods morphology, frequently in pairs or short chains (×1,000). **(D)** The growth curve of LB-Y-1. **(E)** The phylogenetic tree analysis of LB-Y-1. **(F)** Starch-degradation ability. **(G)** Triglyceride-degradation ability.

The colony morphologies of LB-Y-1 was wet, opaque, wrinkled, and irregular on the edges on the surface of LB agar, which was confirmed as gram-positive cocci (elongate oval) by microscopic evaluation (Figures 3A,B). The cells appear frequently in pairs or short chains (Figure 3C). These features suggested that it was related to *Bacillus* spp. The proliferation curves appeared as typically sigmoidal shape, consisting of latency phase (0–6 h), logarithmic phase (6–20 h), and plateau phase (20 h later; Figure 3D). Furthermore, 16S rRNA gene sequencing and phylogenetic analysis found the LB-Y-1 share 100% similarity with the sequences of *B. velezensis* BCRC-17467^T^ (Figure 3E). Biochemical analysis revealed that the main physiological and biochemical characteristics of the LB-Y-1 were similar to *B. velezensis* WLYS23 (Supplementary Table S2). Collectively, LB-Y-1 was identified as *B. velezensis* and deposited it in the China General Microbiological Culture Collection Center (CGMCC, Beijing, China) with accession number 21344. Furthermore, the LB-Y-1 formed hydrolytic circles on the media in the assays for the degradation of starch and triglyceride (Figures 3F,G), indicating that the strain also has the capacity to produce amylase and lipase.

### 3.5. Safety evaluation of LB-Y-1 (*in vitro*)

The hemolytic activity of LB-Y-1 was judged by observing the hemolytic rings on blood agar plates after an 24 h incubation. The strain was not involved in the lysis of erythrocytes (results not shown). Supplementary Table S3 showed the antibiotic susceptibility profile of the LB-Y-1. It was susceptible to 24 routinely used antimicrobials, indicating that the strain is safe and can be used as a probiotic.

### 3.6. *In vivo* testing

#### 3.6.1. Growth performance

**Figure 4:**
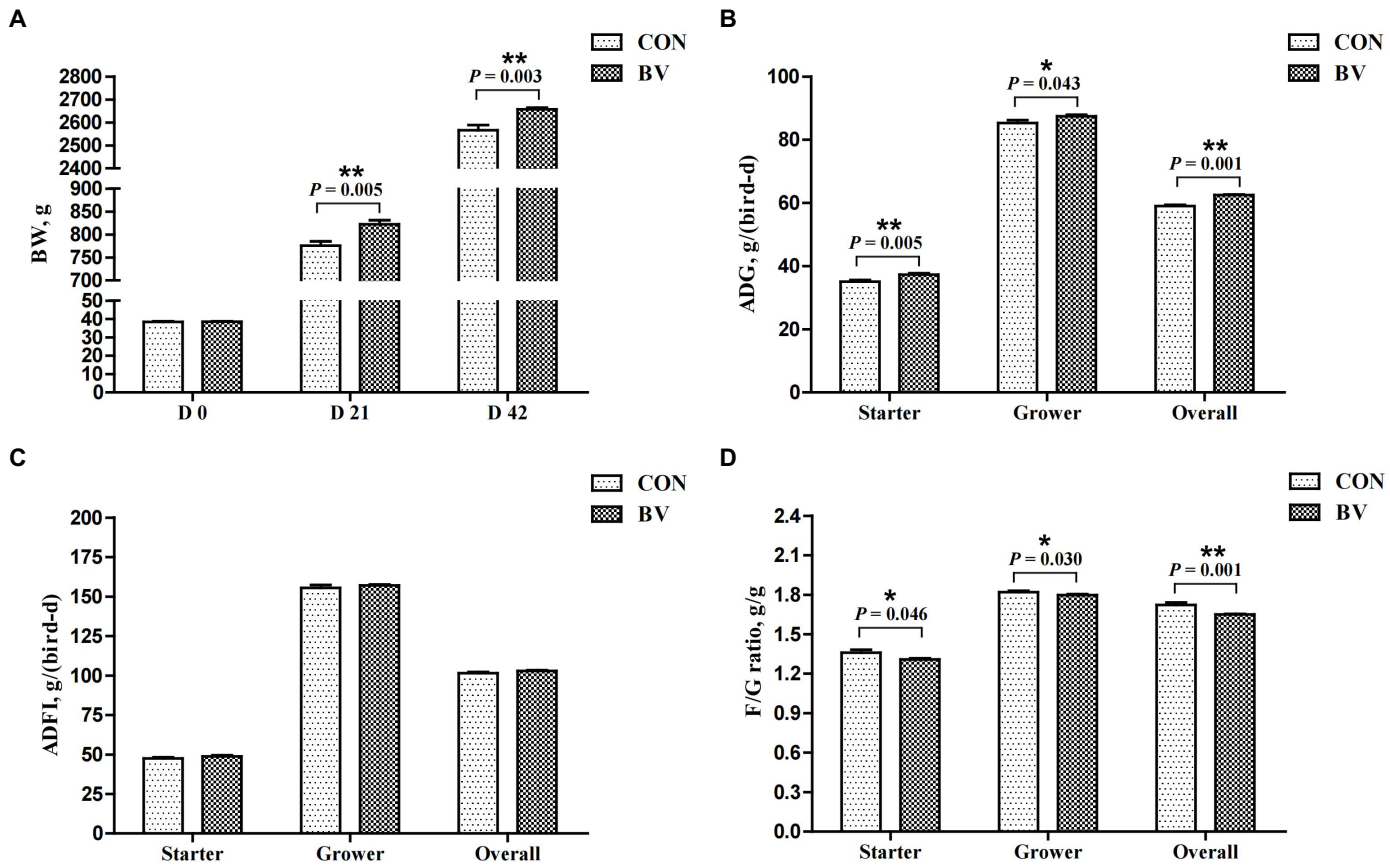
Effect of *Bacillus velezensis* LB-Y-1 on growth performance of broilers (*n* = 6). CON = control group, broilers were fed a corn-soybean basal diet, BV = *B. velezensis* LB-Y-1 group, broilers were fed a basal diet containing 3.5 × 10^9^ CFU/kg LB-Y-1. BW, body weight; ADG, average daily gain; ADFI, average daily feed intake; F/G ratio = feed intake (g)/weight gain (g); Values are presented as Mean ± SEM, the values having superscript (*) were significantly different, *0.01 < *p* < 0.05; **p ≤ 0.01.

The average mortality rate was 0.5% during the experiment (data not presented) with no significant difference between the groups. Growth performance is depicted in Figure 4. Compared with CON group, LB-Y-1 significantly increased the BW of broilers at 21 and 42 days of age, and increased the ADG during the starter, grower, and overall periods (*p* < 0.05). The F/G ratio for the BV group was lower than the CON group during the whole period (*p* < 0.05). No significant differences were found in the ADFI between the groups.

#### 3.6.2. Slaughter performance and immune organ indexes

The characteristics of slaughter performance is presented in Table 1, no significant difference was detected between the groups. In addition, supplementation with LB-Y-1 had no effect on immune organ indexes at 21 and 42 days of age compared to the CON group (Table 2).

**Table 1:** Effect of *Bacillus velezensis* LB-Y-1 on slaughter performance of broilers at day 42 (*n* = 6).

Items	CON	BV	SEM	*p*-value
Dressing percentage, %	88.73	90.03	0.363	0.556
Half-eviscerated yield, %	84.92	84.84	0.219	0.975
Eviscerated yield, %	77.59	76.72	0.363	0.596
Breast muscle ratio, %	13.03	13.10	0.303	0.383
Thigh muscle ratio, %	11.03	11.22	0.580	0.856
Abdominal fat ratio, %	2.41	2.11	0.038	0.970

CON = control group, broiler chickens were fed a corn-soybean basal diet, BV = *B. velezensis* group, broiler chickens were fed a basal diet containing 3.5 × 10^9^ CFU/kg *B. velezensis*. SEM, standard error of means.

**Table 2:** Effect of *Bacillus velezensis* LB-Y-1 on immune organ indexes of broilers (*n* = 6).

Items	Dietary treatment		SEM	*P-*value
	CON	BV		
**Day 21**				
Spleen index, mg/g	0.91	0.89	0.029	0.896
Thymus index, mg/g	2.86	2.84	0.021	0.709
Bursa of fabricius index, mg/g	1.71	1.74	0.020	0.646
**Day 42**				
Spleen index, mg/g	1.20	1.21	0.041	0.649
Thymus index, mg/g	2.15	2.16	0.015	0.789
Bursa of fabricius index, mg/g	1.13	1.19	0.016	0.146

CON = control group, broiler chickens were fed a corn-soybean basal diet, BV = *B. velezensis* group, broiler chickens were fed a basal diet containing 3.5 × 10^9^ CFU/kg *B. velezensis*. Spleen index, thymus index, and bursa of fabricius index were calculated as follows: (1) Spleen index (mg/g) = spleen weight (mg)/body weight (mg), (2) Thymus index (mg/g) = thymus weight (mg)/body weight (g), (3) Bursa of fabricius index (mg/g) = bursa of fabricius (mg)/body weight (g). SEM, standard error of means.

#### 3.6.3. Hematological and serum biochemical indexes analysis

As shown in Table 3, LB-Y-1 treatment significantly increased the levels of ALP (*p* = 0.011, 21 days of age and *p* = 0.007 42 days of age), TP (*p* = 0.001, 21 days of age), Albumin (*p* = 0.002, 21 days of age), A/G ratio (*p* = 0.031, 21 days of age) and P (*p* = 0.001, 42 days of age) compared to the CON group.

**Table 3:** Effect of *Bacillus velezensis* LB-Y-1 on hematological and serum biochemical indexes of broilers (*n* = 6).

Items	Dietary treatment		SEM	*P-*value
	CON	BV		
**Day 21**				
RBC, ×10^12^/L	2.59	2.64	0.083	0.197
HGB, g/L	101.24	102.83	3.126	0.766
WBC, ×10^9^/L	141.57	139.61	1.593	0.097
LYM, ×10^9^/L	55.01	55.72	1.020	0.162
ALT, U/L	2.31	2.19	0.017	0.356
AST, U/L	243.67	241.05	1.207	0.609
ALP, U/L	2872.67	2960.17^*^	18.851	0.011
Phosphorus, mmol/L	1.50	1.54	0.011	0.065
TP, g/L	3.09	3.26^**^	0.021	0.001
Albumin, g/L	1.34	1.48^**^	0.018	0.002
Globulin, g/L	1.75	1.78	0.010	0.297
A/G ratio	0.77	0.83^*^	0.010	0.031
**Day 42**				
RBC, ×10^12^/L	2.47	2.50	0.080	0.497
HGB, g/L	100.53	99.67	3.372	0.441
WBC, ×10^9^/L	142.52	139.35	1.354	0.085
LYM, ×10^9^/L	57.38	59.20	0.946	0.246
ALT, U/L	3.25	3.17	0.020	0.082
AST, U/L	239.85	232.43	1.343	0.185
ALP, U/L	2789.83	2880.67^**^	18.831	0.007
Phosphorus, mmol/L	1.45	1.53^*^	0.016	0.001
TP, g/L	3.15	3.22	0.026	0.159
Albumin, g/L	1.39	1.43	0.016	0.095
Globulin, g/L	1.76	1.79	0.013	0.446
A/G ratio	0.79	0.80	0.007	0.274

CON = control group, broiler chickens were fed a corn-soybean basal diet, BV = *B. velezensis* group, broiler chickens were fed a basal diet containing 3.5 × 10^9^ CFU/kg *B. velezensis*. RBC, red blood cell count; HGB, hemoglobin concentration; WBC, white blood cell count; Lym, lymphocytes; ALT, alanine aminotransferase; AST, aspartate aminotransferase; TP, total protein; A/G ratio = albumin/globulin; SEM, standard error of means. ^*^P < 0.05 and ^**^P < 0.01 indicate a significant difference within row.

#### 3.6.4. Intestinal digestive enzyme

The effect of LB-Y-1 on intestinal digestive enzyme of broilers is depicted in Table 4. Broilers with LB-Y-1 had higher amylase and trypsin activity at 21 days of age and higher amylase activity at 42 days of age compared to the CON group (*p* < 0.05).

**Table 4:** Effect of *Bacillus velezensis* LB-Y-1 on digestive enzyme activity of broilers (*n* = 6).

Items	Dietary treatment		SEM	*P-*value
	CON	BV		
**Day 21**				
Amylase (U/mg protein)	174.17	205.08^**^	5.709	0.001
Trypsin (U/mg protein)	4374.04	4621.04^**^	48.410	0.003
Lipase (U/g protein)	26.51	26.70	0.174	0.626
**Day 42**				
Amylase (U/mg protein)	123.08	144.75^*^	4.861	0.017
Trypsin (U/mg protein)	4758.05	4897.69	53.050	0.202
Lipase (U/g protein)	23.28	22.47	0.355	0.271

CON = control group, broiler chickens were fed a corn-soybean basal diet, BV = *B. velezensis* group, broiler chickens were fed a basal diet containing 3.5 × 10^9^ CFU/kg *B. velezensis*. SEM, standard error of means. ^*^P < 0.05 and ^**^P < 0.01 indicate a significant difference within row.

#### 3.6.5. Tibia bone mineralization

**Figure 5:**
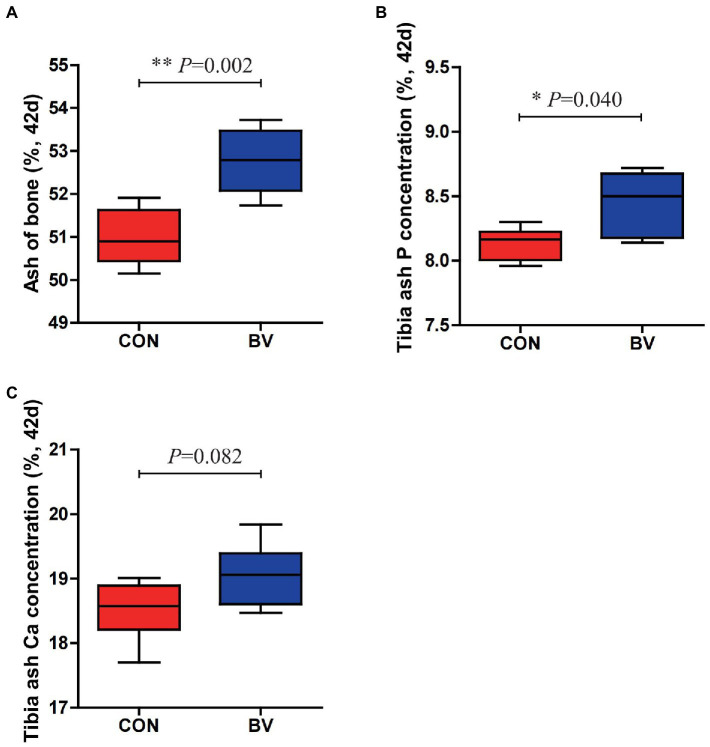
Effect of *Bacillus velezensis* LB-Y-1 on tibia bone mineralization of broilers (*n* = 6). **(A)** Bone ash content at 42 days of age. **(B)** Tibia ash P concentration at 42 days of age. **(C)** Tibia ash Ca concentration at 42 days of age. Values are presented as Mean ± SEM, the values having superscript (*) were significantly different, *p < 0.05; **p < 0.01.

The effect of LB-Y-1 on tibia bone mineralization of broilers is depicted in Figure 5. Supplementation with LB-Y-1 significantly increased tibia bone ash at 42 days of age, along with tibia ash P concentration compared to control broilers (*p* < 0.05). Although not significant, tibia ash Ca concentration was also elevated in the BV group relative to broilers of CON group.

#### 3.6.6. Histological analysis

Table 5 showed the effect of LB-Y-1 on intestinal histomorphology of broilers. LB-Y-1 significantly decreased the crypt depth of jejunum, and increased the ratio of villus height to crypt depth at 21 days of age (*p* < 0.05). There were no significant differences in histomorphometric features of the jejunum and ileum between the CON and BV groups at 42 days of age. Furthermore, LB-Y-1 had no significant effect on epithelial thickness of jejunum and ileum at 21 and 42 days of age.

**Table 5:** Effect of *Bacillus velezensis* LB-Y-1 on histomorphology of the small intestinal sections in broilers (*n* = 6).

Items	Intestine	Treatment		SEM	*P-*value
		CON	BV		
**Day 21**					
Jejunum	Villus length, μm	1259.88	1164.63	28.512	0.095
	Crypt depth, μm	183.65^**^	153.32	5.746	0.002
	V/C ratio	6.86	7.62^**^	0.174	0.021
	Epithelial thickness, μm	218.15	227.98	6.046	0.443
Ileum	Villus length, μm	805.98	773.19	12.126	0.188
	Crypt depth, μm	197.87	186.35	4.258	0.187
	V/C ratio	4.09	4.15	0.087	0.747
	Epithelial thickness, μm	224.77	214.89	5.246	0.371
**Day 42**					
Jejunum	Villus length, μm	1295.59	1210.03	27.473	0.123
	Crypt depth, μm	213.20	210.51	4.932	0.799
	V/C ratio	6.09	5.77	0.126	0.219
	Epithelial thickness, μm	279.57	255.17	7.758	0.119
Ileum	Villus length, μm	831.92	818.94	6.493	0.341
	Crypt depth, μm	209.55	192.72	4.900	0.085
	V/C ratio	3.99	4.27	0.095	0.153
	Epithelial thickness, μm	346.03	325.97	10.154	0.347

CON = control group, broiler chickens were fed a corn-soybean basal diet, BV = *B. velezensis* group, broiler chickens were fed a basal diet containing 3.5 × 10^9^ CFU/kg *B. velezensis*. V/C ratio = Villus length (μm)/Crypt depth (μm). SEM, standard error of means. ^*^P < 0.05 and *P* < 0.01 indicate a significant difference within row.

#### 3.6.7. Microbial analysis

**Figure 6:**
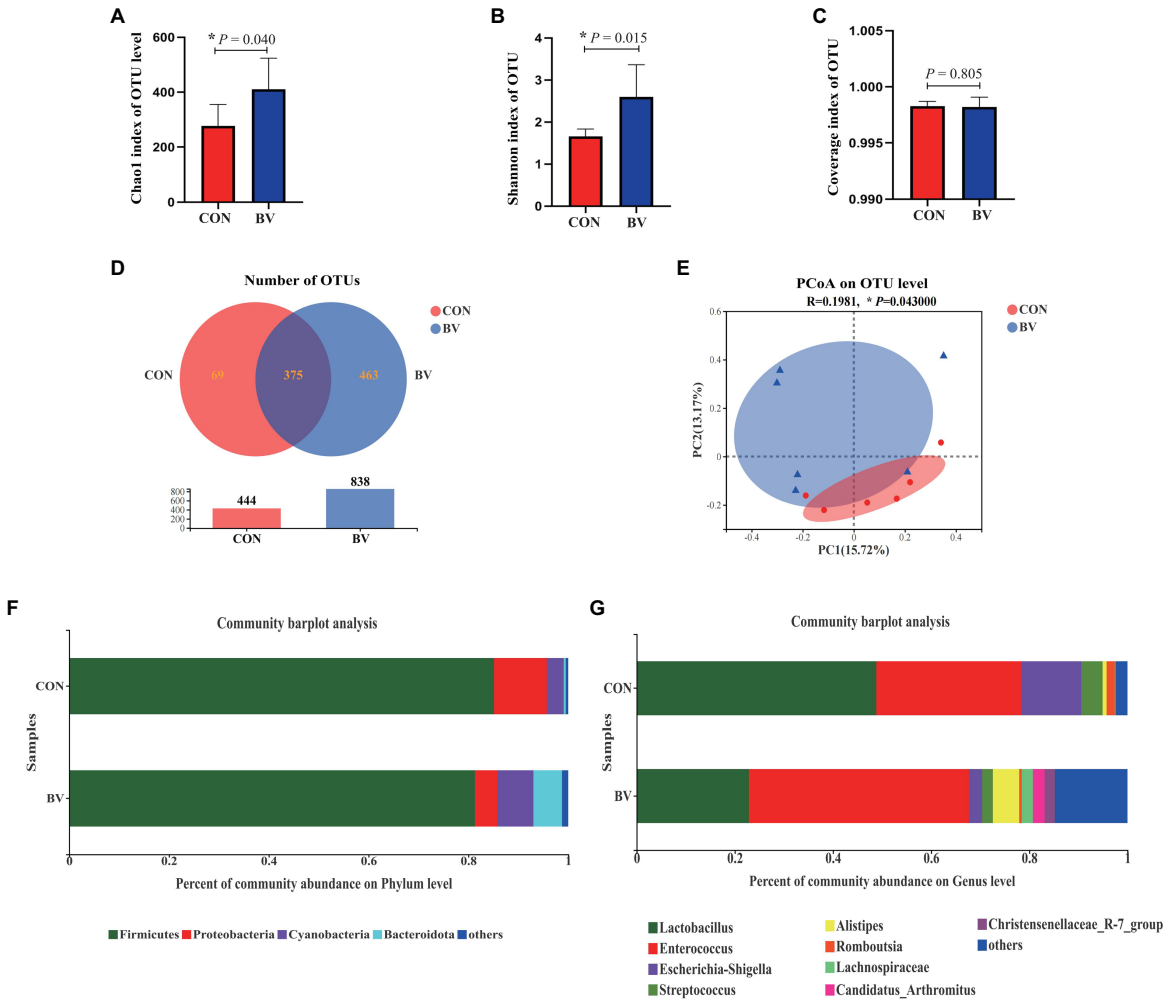
Effect of *Bacillus velezensis* LB-Y-1 on the ileal microbial diversity of broilers (*n* = 6). **(A)** Chao1 index of OUT level. **(B)** Shannon index of OUT level. **(C)** Coverage index of OUT level. **(D)** Number of OTUs. **(E)** β-diversity was estimated by the PCoA on OUT level. **(F,G)** The relative abundance of bacteria at the phylum and genus levels, respectively. Values are presented as Mean ± SEM, the values having superscript (*) were significantly different, *0.01 < *p* < 0.05; **p ≤ 0.01.

**Figure 7:**
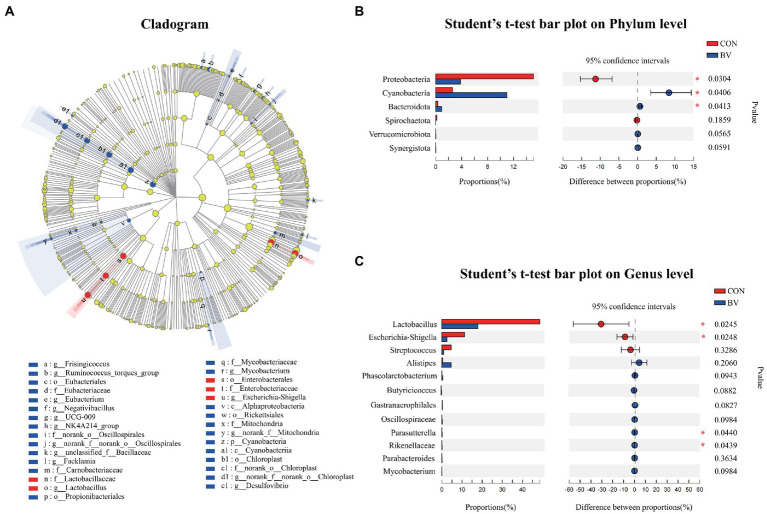
Effect of *Bacillus velezensis* LB-Y-1 on the abundance of the ileal microbial community of broilers (*n* = 6). **(A)** Cladogram of LEfSe multilevel species difference discriminant analysis (LDA > 2), different color nodes indicate microbial communities that are significantly enriched in the corresponding groups and significantly different between groups. **(B,C)** Comparative analysis of the relative abundance of bacteria at the phylum and genus levels, respectively. Values are presented as Mean ± SEM, the values having superscript (*) were significantly different, *0.01 < *p* < 0.05; **p ≤ 0.01.

To understand whether LB-Y-1 could modulate gut microbiota community, we investigated the change of the ileal microbiota diversity. A community richness (Chao1 index) and diversity (Shannon index) analysis demonstrated that broilers supplemented with the LB-Y-1 had significantly higher microbial relative abundance and potential diversity than those of CON group (*p* < 0.05; Figures 6A,B). The coverage index was greater than 0.998 in each group (Figure 6C), indicating an adequate depth of sequencing. Furthermore, a higher OTU richness was observed in the BV group (Figure 6D). The *β*-diversity analysis indicated that samples in CON and BV groups had different community composition and structure, suggesting a significant segregation of microbiota between the groups (Figure 6E). *Firmicutes* was the predominant phyla (Figure 6F), and *Lactobacillus* and *Enterococcus* were the main dominant genera (Figure 6G). The LEfSe analysis (LDA > 2) revealed the significant differences in microbiota structure between the CON group and the BV group (Figure 7A). Student’s *t*-test showed that BV group had a lower abundance of *Proteobacteria*, and higher abundance of *Cyanobacteria* and *Bacteroidota* at phylum level (*p* < 0.05, Figure 7B); as well as BV group had lower abundance of *Lactobacillus* and *Escherichia-Shigella*, and higher abundance of *Parasutterella* and *Rikenellacea*e at genus level (*p* < 0.05, Figure 7C).

## 4. Discussion

*Bacillus* spp. is emerging as a promising probiotic candidate because of the following characteristics: (1) it has the ability to form as spores and has less loss during feed preparation; (2) it is easy to produce by batch (Hyronimus et al., 2000). Furthermore, *Bacillus* spp. is recognized an excellent probiotic that produces multi-enzyme, bacteriocin, and beneficial metabolites (Li et al., 2018). Previous studies have also shown positive effects of *Bacillus* spp. on nutrient utilization, intestinal integrity, and growth performance of broilers (Salim et al., 2013; Rivera-Pérez et al., 2021). Due to the shortage of traditional feed and the rapid rise in the price of protein raw materials, it is particularly important to improve the utilization of protein in feed, which prompted us to screen a strain of *Bacillus* spp. that could exert a positive effect on poultry production.

It is known that incomplete digestion of proteins can lead to the multiplication of putrefactive microorganisms and the accumulation of toxic metabolites (Dallas et al., 2017). High levels of protein in excreta are not only a waste of resources, but also harmful to the environment. Based on these, the protease production efficiency of *Bacillus* spp. was used as an evaluation index in the primary screening. Further, unlike mammals and other waterfowl, broilers have lower endogenous cellulase activity, which limits fiber digestion. The addition of exogenous fiber degrading enzymes can significantly reduce chyme viscosity and improve nutrient digestibility (Slominski et al., 2006; Zhai et al., 2020). Therefore, the cellulase production capacity was taken as the evaluation index in the second screening step. There is no doubt that microbial degradation of phytic acid is essential to prevent environmental phosphate contamination and to deal with nutritional problems in monogastric animals. Phytase is widely used to improve the efficiency of phosphate absorption in animal feed (Blüher et al., 2017). In the tertiary screening, the phytase production efficiency of *Bacillus* spp. was taken as an evaluation index. Eventually, the strain of LB-Y-1 showed the best performance. According to the morphological and molecular characteristics, we preliminary identified the target strain as *B. velezensis*. Biochemical analysis revealed that, the main physiological and biochemical characteristics of the LB-Y-1 were similar to *B. velezensis* WLYS23, which has been reported (Zhang D. F. et al., 2021). However, unlike the WLYS23, the LB-Y-1 did not react with inulin, but it was consistent with another standard strain of *B. velezensis* CR-502^T^ (Ruiz-García et al., 2005). The safety evaluation of LB-Y-1 indicated that LB-Y-1 was not haemolytic and highly sensitive to common antibiotics. Furthermore, consistent with the previous report (Li et al., 2018), we too found that the LB-Y-1 had the potential to degrade starch and triglyceride components. These results indicate that LB-Y-1 has potential as a safety probiotic candidate.

Like mammals, digestive system of poultry is underdeveloped early in life and cannot secrete enough gastric acid and digestive enzymes (Zhang et al., 2022). In addition, poultry has a limited ability to digest fiber in feed compared with mammals, and high-fiber or slowly digestible protein diets will cause loss of production performance (Berrocoso et al., 2020; Zhang Y. C. et al., 2021). Fortunately, it was found that the addition of exogenous enzymes may help to solve this problem (Tavernari et al., 2008). Our study found that adding LB-Y-1 increased BW and feed conversion of broilers without affecting feed intake, which we linked to the properties of LB-Y-1 secreting multi-enzyme, and this positive effect was consistent with the results of other study (Wang et al., 2021). The effect of intestinal digestive enzymes appear in the response of *Bacillus* spp. in two ways. On the one hand, the members of *Bacillus* spp. can secrete a variety of extracellular enzymes. On the other hand, *Bacillus* spp. can stimulate the secretion of endogenous enzymes and regulate the gut microbial flora (Bedford and Schulze, 1998). Our study indicated that the cellulase and phytase secreted by LB-Y-1 contribute to the release of nutrients in feed, the protease and amylase secreted by LB-Y-1 enhanced the intestinal digestibility, thus promoting the digestion of nutrients. It has also been shown that *Bacillus* spp. may contribute to improved morphology of intestine by increasing villus height and ratio of villus height to crypt depth (Mohamed et al., 2022). The results are consistent with our findings, LB-Y-1 improved the intestinal structure at an early stage, which may contribute to the gut health.

Study in broilers demonstrated that dietary supplementation with *Bacillus* spp. improved mineralization of tibial (Latorre et al., 2017). In the current study, ALP activity was increased when LB-Y-1 were added to the diet. ALP is a kind of enzyme involved in phosphate hydrolysis and a marker of skeletal mineralization in broilers (Tilgar et al., 2008; Escobar et al., 2022). The increased activity of ALP was accompanied by a significant increase in bone phosphorus, in addition, the level of phosphorus of broilers fed LB-Y-1 was also significantly increased, which had been proved by Li et al. with *B. amyloliquefaciens* (Li et al., 2022). In addition, the phytase produced by LB-Y-1 can also promote the release of phosphorus from phytate complex in the feed (Viveros et al., 2002), thus improving the phosphorus utilization. These results indicated that LB-Y-1 could promote the metabolism and utilization of phosphorus.

It has previously been shown that the diversity of gut microbes contributes to microbiome homeostasis and the resistance to pathogenic microorganisms (Konstantinov et al., 2004). Besides this, the intestinal microbiota community plays a variety of roles in nutrient absorption and metabolism, immunity, as well as bone density and strength (Bielke et al., 2017; Hong et al., 2019). In this study, dietary supplementation with LB-Y-1 increased the intestinal microbial diversity (Shannon index) and the microbial community richness (Chao1 index). The PCoA showed obviously different pattern of microbial communities between the groups. The predominant bacterial phylum of ileum include *Firmicutes*, *Cyanobacteria*, *Proteobacteria*, and *Bacteroidota* (Guo et al., 2021), our study observed that LB-Y-1 increased the abundance of *Cyanobacteria* and *Bacteroidota*, and decreased the abundance of *Proteobacteria*. *Cyanobacteria* is the dominant phyla in the healthy intestinal tract of mammals and poultry, which can play the role of nitrogen fixation and also contribute to the nutrient absorption in the intestines (Mandal et al., 2016; Yang et al., 2020), so the increased abundance of *Cyanobacteria* indicated that LB-Y-1 could improve the structure of the flora. *Bacteroidota* has been extensively studied for its regulatory effect on the host, which can accelerate angiogenesis in the intestinal mucosa, enhance the host’s immunity, and maintain the balance of intestinal microbiota (Cheng et al., 2022). It is well known that *Proteobacteria* contains a wide variety of pathogens such as *Salmonella*, *Escherichia coli*, and *Shigella*, which could exert pathogenic effects in the intestine of broilers (Mora et al., 2010). Therefore, the decrease of *Proteobacteria* in BV group indicated that a relatively healthy bacterial community was achieved by LB-Y-1 supplementation. Furthermore, the results of the analysis at the genus level reinforced this conclusion. *Escherichia-Shigella* with reduced relative abundance in our study is an opportunistic pathogen that is positively correlated with a variety of intestinal infections (Yang et al., 2019). Correspondingly, we also found an increase in the abundance of some beneficial genera, such as *Parasutterella* and *Rikenellaceae. Parasutterella* is an important player in multiple gastrointestinal metabolic processes, which has been shown to have a positive role in tyrosine, cholesterol and bile acid metabolism (Ju et al., 2019). *Rikenellaceae* plays an important role in promoting the fermentation of carbohydrates and proteins *in vivo*, and can reduce the damage of intestinal immune function and the occurrence of intestinal inflammation (Su et al., 2014; Donaldson et al., 2016). For the role in bone development, Li′s report suggests that *Bacillus* spp. can modulate the gut microbiome structure to affect the biosynthesis of polyamines, which in turn can mediate the enhancement of osteoblast activity and have a positive effect on increasing bone strength (Li et al., 2022). Overall, the results revealed that LB-Y-1 can promote the organismal development of broilers by improving the structure of intestinal microbiota.

## 5. Conclusion

The present study demonstrated that the newly screened and characterized *B. velezensis* LB-Y-1, which was isolated from the intestinal tract of different healthy animals, has marked multi-enzyme production property. *In vivo* broilers assay indicated that LB-Y-1 has the potential to improve broiler growth performance and tibia mineralization, the mechanism may be associated with the enhanced intestinal digestive enzyme activities, increased P retention and alterations of intestinal microbiota structure. These results are encouraging and suggesting that *B. velezensis* LB-Y-1 is a potential strain for further utilization in direct-fed microbial or probiotic starter culture.

## Data availability statement

The datasets presented in this study can be found in online repositories. The names of the repository/repositories and accession number(s) can be found in the article/Supplementary material.

## Ethics statement

The animal study was reviewed and approved by Animal Ethics Committee of the Chinese Academy of Agricultural Sciences (AEC-CAAS-20191106, Beijing, China).

## Author contributions

CL, SL, and GD: conceptualization. CL and SL: methodology. CL, GD, and RJ: investigation, software, data curation, and writing-original draft preparation. SC and XD: validation and resources. GL and YB: writing review, editing, and supervision. HC: project administration and funding acquisition. All authors contributed to the article and approved the submitted version.

## Funding

This research was funded by China Agriculture Research System (CARS-41).

## Conflict of interest

The authors declare that the research was conducted in the absence of any commercial or financial relationships that could be construed as a potential conflict of interest.

## Publisher’s note

All claims expressed in this article are solely those of the authors and do not necessarily represent those of their affiliated organizations, or those of the publisher, the editors and the reviewers. Any product that may be evaluated in this article, or claim that may be made by its manufacturer, is not guaranteed or endorsed by the publisher.

## Footnotes

^1^ www.megasoftware.net

^2^ https://cloud.majorbio.com/
