# Supplementary material for: Screening and characterization of Bacillus velezensis LB-Y-1 toward selection as a potential probiotic for poultry with multi-enzyme production property
Source: Front Microbiol. 2023 Apr 17;14:1143265. doi: 10.3389/fmicb.2023.1143265 (PMC10149742; doi:10.3389/fmicb.2023.1143265)
Supplement: Supplementary file 1 [file Table_1.DOCX]

Supplementary Material

Screening and Characterization of *Bacillus velezensis* LB-Y-1 Toward the Selection as a Potential Probiotic for Poultry with Multi-enzyme Production Property

Chong Li^1,2†^, Shuzhen Li^1†^, Guoqi Dang^2†^, Rui Jia^1^, Si Chen^3^, Xuejuan Deng^4^, Guohua Liu^1^, Yves Beckers^2^, Huiyi Cai^1,4*^

^1^ Key Laboratory for Feed Biotechnology of the Ministry of Agriculture and Rural Affairs, Institute of Feed Research, Chinese Academy of Agriculture Sciences, Beijing 100081, China

^2^ Precision Livestock and Nutrition Laboratory, Teaching and Research Centre (TERRA), Gembloux Agro-Bio Tech, University of Liège, Gembloux 5030, Belgium

^3^ Department of Molecular Cell Biology, Samsung Medical Center, Sungkyunkwan University School of Medicine, Suwon, South Korea

^4^ National Engineering Research Center of Biological Feed, Beijing 100081, China

^†^ These authors have contributed equally to this work

*** Correspondence:** Corresponding Author: Huiyi Cai [caihuiyi@caas.cn](mailto:caihuiyi@caas.cn)

**Supplementary File 1:**

**Molecular biological identification of LB-Y-1**

The genomic DNA of strain LB-Y-1 was extracted using the bacterial DNA extraction kit, and then the 16S rRNA and gyrB gene were amplified by PCR using the methods described by (Sun et al., 2016; Zhang et al., 2016):

The bacterial 16S rRNA was amplified using universal primers:

27F (5'-AGAGTTTGATCMTGGCTCAG-3')

1492R (5'-TACGGYTACCTTGTTACGACTT-3') were used for amplification of the 16S rRNA gene;

Primers used for gyrB gene:

UP1 (5'-GAAGTCATCATGACCGTTCTGCAYGCNGGNGGNAARTTYGA-3')

UP2r (5'-AGCAGGGTACGGATGTGCGAGCCRTCNACRTCNGCRTCNGTCAT-3')

The PCR products were sequenced by the Sangon Biotech Co., Ltd. (Shanghai, China). The homologies between the obtained gene sequences and those in GenBank were evaluated using BLAST analysis on the National Center for Biotechnology Information (NCBI).

Sun, J., Zhang, X., Gao, X., Jiang, Q., Wen, Y., and Lin, L. (2016). Characterization of Virulence Properties of Aeromonas veronii Isolated from Diseased Gibel Carp (Carassiusgibelio). *Int. J. Mol. Sci.* 17, 1–11. doi:10.3390/IJMS17040496.

Zhang, B., Wang, Y., Tan, Z., Li, Z., Jiao, Z., and Huang, Q. (2016). Screening of Probiotic Activities of Lactobacilli Strains Isolated from Traditional Tibetan Qula, A Raw Yak Milk Cheese. *Asian-Australasian J. Anim. Sci.* 29, 1490. doi:10.5713/AJAS.15.0849.

**Supplementary File 2:**

**Table S1.** Analysis composition of basal diets and nutrient level (air-dry basis, %).

| **Ingredient (g/kg)** | **Starter** |  | **Grower** |
| --- | --- | --- | --- |
|  | **(1–21 days)** |  | **(22–42 days)** |
| Corn | 557.3 |  | 581.5 |
| Soybean meal, (CP 43%) | 309.4 |  | 291.3 |
| Corn gluten meal, (CP 60%) | 58.9 |  | 44.1 |
| Soybean oil | 31.0 |  | 45.7 |
| L-Lysine | 2.4 |  | 1.1 |
| *DL*-Methionine | 2.2 |  | 1.3 |
| Limestone | 13.4 |  | 12.5 |
| CaHPO_4_ | 19.9 |  | 16.9 |
| NaCl | 2.8 |  | 2.8 |
| Choline chloride | 0.2 |  | 0.3 |
| Mineral premix^1)^ | 2.5 |  | 2.5 |
| Total | 1000 |  | 1000 |
| Nutrient concentrations^2)^ |  |  |  |
| Metabolic energy (MJ/kg) | 12.5 |  | 13.0 |
| Crude protein | 21.5 |  | 20.1 |
| Calcium | 10.0 |  | 10.3 |
| Available P | 4.5 |  | 4.0 |
| Total P | 6.9 |  | 6.1 |
| Lysine | 11.5 |  | 10.0 |
| Methionine | 5.0 |  | 4.0 |
| Methionine + Cysteine | 8.1 |  | 6.4 |

^1)^ The premix provided the following per kilogram diet: vitamin A 10, 000 IU, vitamin D_3_ 2000 IU, vitamin E 10 IU, vitamin K_3_ 2.5 mg, vitamin B_1_ 1 mg, vitamin B_2_ 6 mg, vitamin B_3_ 10 mg, vitamin B_5_ 40 mg, vitamin B_6_ 3 mg, vitamin B_11_ 0.3 mg, vitamin B_12_ 0.01 mg, biotin 0.12 mg, Cu (as copper sulfate) 8 mg, Fe (as ferrous sulfate) 80 mg, Mn (as manganese sulfate) 60 mg, Zn (as zinc sulfate) 40 mg, Se (as sodium selenite) 0.15 mg, I (as potassium iodide) 0.35 mg. ^2)^ Calculated nutrient concentrations.

**Supplementary File 3:**

**Table S2.** Biochemical characterization of LB-Y-1, WLYS23 and CR-502^T^ strains.

| **No.** | **Active ingredient** | **Strains** | | |
| --- | --- | --- | --- | --- |
|  |  | **LB-Y-1** | **WLYS23** | **CR-502^T^** |
| 1 | Glycerol | ＋ | ＋ | ＋ |
| 2 | Erythritol | － | － | － |
| 3 | D-arabinose | － | － | － |
| 4 | L-arabinose | ＋ | ＋ | ＋ |
| 5 | D-ribose | ＋ | ＋ | ＋ |
| 6 | D-xylose | ＋ | ＋ | ＋ |
| 7 | L-xylose | － | － | － |
| 8 | D-adonitol | － | － | － |
| 9 | Methyl-β-D-xylopyranoside | － | － | － |
| 10 | D-galactose | － | － | － |
| 11 | D-glucose | ＋ | ＋ | ＋ |
| 12 | D-fructose | ＋ | ＋ | ＋ |
| 13 | D-mannose | ＋ | ＋ | ＋ |
| 14 | D-sorbose | － | － | － |
| 15 | L-rhamnose | － | － | － |
| 16 | Dulcitol | － | － | － |
| 17 | Inositol | － | － | ＋ |
| 18 | D-mannitol | ＋ | ＋ | ＋ |
| 19 | D-sorbitol | ＋ | ＋ | ＋ |
| 20 | Methyl-D-mannopyranoside | － | － | － |
| 21 | Methyl-D-glucopyranoside | ＋ | ＋ | ＋ |
| 22 | N-acetylglucosamine | ＋ | ＋ | － |
| 23 | Amygdalin | ＋ | ＋ | ＋ |
| 24 | Arbutin | ＋ | ＋ | ＋ |
| 25 | Esculin ferric citrate | ＋ | ＋ | ＋ |
| 26 | Salicin | ＋ | ＋ | ＋ |
| 27 | D-cellobiose | ＋ | ＋ | ＋ |
| 28 | D-maltose | ＋ | ＋ | ＋ |
| 29 | D-lactose (bovine origin) | ＋ | ＋ | ＋ |
| 30 | D-melibiose | － | － | － |
| 31 | D-saccharose | ＋ | ＋ | ＋ |
| 32 | D-trehalose | ＋ | ＋ | ＋ |
| 33 | Inulin | － | ＋ | － |
| 34 | D-melezitose | － | － | － |
| 35 | D-raffinose | ＋ | ＋ | ＋ |
| 36 | Amidon (starch) | ＋ | ＋ | ＋ |
| 37 | Glycogen | ＋ | ＋ | ＋ |
| 38 | Xylitol | － | － | － |
| 39 | Gentiobiose | ＋ | ＋ | ＋ |
| 40 | D-turanose | － | － | － |
| 41 | D-lyxose | － | － | － |
| 42 | D-tagatose | － | － | － |
| 43 | D-fucose | － | － | － |
| 44 | L-fucose | － | － | － |
| 45 | D-arabitol | － | － | － |
| 46 | L-arabitol | － | － | ＋ |
| 47 | Potassium gluconate | － | － | － |
| 48 | Potassium 2-ketogluconate | － | － | － |
| 49 | Potassium 5-ketogluconate | － | － | － |
| 50 | Vogues-Proskauer | － | － | ＋ |
| 51 | citrate | － | － | － |
| 52 | propionate | － | － | ND |
| 53 | gelatin | ＋ | ＋ | ＋ |
| 54 | 7% NaCl | － | － | ＋ |
| 55 | pH5.7 | ＋ | ＋ | ＋ |
| 56 | nitrate reduction | ＋ | ＋ | ＋ |

“－” Represents the bacterium does not use this carbohydrate. “＋” Represents the bacterium uses this carbohydrate; No. 1-49, the tests were carried out using API 50 CHB (bioMérieux); No. 50-56, the tests were carried out using HBI Bacillus identification strips (Qingdao Hope Bio-Technology Co., Ltd, Qingdao, China).

**Supplementary File 4:**

**Table S3.** Antibiotic susceptibility profile of potential probiotic *B. velezensis* LB-Y-1.

| **Drug** | **Content (μg/tablet)** | **Susceptibility^a^** | **Drug** | **Content (μg/tablet)** | **Susceptibility^a^** |
| --- | --- | --- | --- | --- | --- |
| Cefoperazone | 75 | S | Erythromycin | 15 | S |
| Ceftriaxone | 30 | S | Neomycin | 30 | S |
| Ceftazidime | 30 | S | Kanamycin | 30 | S |
| Cefuroxime | 30 | S | Gentamicin | 10 | S |
| Cefradine | 30 | S | Amikacin | 30 | S |
| Cefazolin | 30 | S | Vancomycin | 30 | S |
| Cefalexin | 30 | S | Piperacillin | 100 | S |
| Minocyline | 30 | S | Ampicillin | 100 | S |
| Doxycycline | 30 | S | Oxacillin | 1 | S |
| Tetracycline | 30 | S | Penicillin | 10 | S |
| Ciprofloxacin | 5 | S | Chloramphenicol | 30 | S |
| Clindamycin | 2 | S | Furazolidone | 300 | S |

^a^ Susceptibility expressed as S=Sensitive, I=Intermediate and R=Resistance.
